# Declines in Outpatient Antimicrobial Use in Canada (1995–2010)

**DOI:** 10.1371/journal.pone.0076398

**Published:** 2013-10-16

**Authors:** Rita Finley, Shiona K. Glass-Kaastra, Jim Hutchinson, David M. Patrick, Karl Weiss, John Conly

**Affiliations:** 1 Centre for Food-borne, Environmental and Zoonotic Infectious Diseases, Public Health Agency of Canada, Guelph, Ontario, Canada; 2 Division of Medical Microbiology, Island Medical Program, University of British Columbia, Victoria, British Columbia, Canada; 3 British Columbia Centre for Disease Control, Vancouver, British Columbia, Canada; 4 School of Population and Public Health, University of British Columbia, Vancouver, British Columbia, Canada; 5 Department of Infectious Diseases and Microbiology, Hôpital Maisonneuve-Rosemont, University of Montreal, Montreal, Québec, Canada; 6 Department of Medicine, University of Calgary, Calgary, Alberta, Canada; 7 Department of Microbiology, Immunology and Infectious Diseases, University of Calgary, Calgary, Alberta, Canada; 8 Department of Pathology and Laboratory Medicine, University of Calgary, Calgary, Alberta, Canada; 9 Synder Institute for Chronic Diseases, University of Calgary, Calgary, Alberta, Canada; Rockefeller University, United States of America

## Abstract

**Background:**

With rising reports of antimicrobial resistance in outpatient communities, surveillance of antimicrobial use is imperative for supporting stewardship programs. The primary objective of this article is to assess the levels of antimicrobial use in Canada over time.

**Methods:**

Canadian antimicrobial use data from 1995 to 2010 were acquired and assessed by four metrics: population-adjusted prescriptions, Defined Daily Doses, spending on antimicrobials (inflation-adjusted), and average Defined Daily Doses per prescription. Linear mixed models were built to assess significant differences among years and antimicrobial groups, and to account for repeated measurements over time. Measures were also compared to published reports from European countries.

**Results:**

Temporal trends in antimicrobial use in Canada vary by metric and antimicrobial grouping. Overall reductions were seen for inflation-adjusted spending, population-adjusted prescription rates and Defined Daily Doses, and increases were observed for the average number of Defined Daily Doses per prescription. The population-adjusted prescription and Defined Daily Doses values for 2009 were comparable to those reported by many European countries, while the average Defined Daily Dose per prescription for Canada ranked high. A significant reduction in the use of broad spectrum penicillins occurred between 1995 and 2004, coupled with increases in macrolide and quinolone use, suggesting that replacement of antimicrobial drugs may occur as new products arrive on the market.

**Conclusions:**

There have been modest decreases of antimicrobial use in Canada over the past 15 years. However, continued surveillance of antimicrobial use coupled with data detailing antimicrobial resistance within bacterial pathogens affecting human populations is critical for targeting interventions and maintaining the effectiveness of these products for future generations.

## Introduction

Concern over the development and spread of antimicrobial resistance (AMR) has dramatically changed our way of thinking about antimicrobial use over the last 70 years. When antimicrobials were first marketed, mortality due to infectious diseases dropped dramatically, ushering in a new era of medical treatment. However, the consequences of relatively unregulated use were not predicted and selection pressure for antimicrobial resistance increased with use. Until the 1980s, new antimicrobials were being discovered and marketed which allowed for the treatment of organisms resistant to older antimicrobials [1]. In contrast, during the 1990s and 2000s, the “pipeline” of new antimicrobial development became stagnant, limiting treatment options for infections caused by pathogens harbouring resistance. In the face of this challenge, efforts to combat AMR have shifted to antimicrobial stewardship in an effort to decrease selection pressure for AMR rather than the development of new agents to treat resistant organisms [2], [3].

Canadian policy makers, researchers, and physicians have been committed to antimicrobial stewardship, dating back to 1997, following a Consensus Conference entitled “Controlling Antimicrobial Resistance: an Integrated Action Plan for Canada” [4]–[8]. Past monitoring of antimicrobial use in Canada was provided by the Canadian Committee for Antimicrobial Resistance, which used data acquired by IMS Health Canada [1]. Currently, the Canadian Integrated Program for Antimicrobial Resistance Surveillance (CIPARS), coordinated by the Public Health Agency of Canada (PHAC), is the only national program systematically monitoring the use of oral antimicrobial drugs, an essential component of a complete system for AMR surveillance. CIPARS tracks temporal and regional trends in antimicrobial use and antimicrobial resistance in selected species of enteric bacteria obtained at different stages of food production and from human clinical laboratory submissions [9]. Data monitored as part of the human antimicrobial use component includes prescriptions dispensed through community pharmacies, purchases of antimicrobials by hospitals, and diagnoses for which physicians have recommended antimicrobials; all of these data are acquired from IMS Health Canada.

The primary objective of this paper was to assess overall trends in human out-patient oral antimicrobial use in Canada using four measures of consumption, and trends in eight groupings of antimicrobials over time. A secondary objective was to compare antimicrobial use measures from Canada to those published by European countries through the European Surveillance of Antimicrobial Consumption Network (ESAC-Net) [10].

## Methods

### Data Collection and Antimicrobial Classification

The Canadian CompuScript (CSC) dataset (2000–2010) was obtained from IMS Health Canada (http://www.imshealth.com) by PHAC-CIPARS. In addition, supplementary data also collected by IMS Health Canada were provided to the former Canadian Committee for Antimicrobial Resistance (CCAR), which was active from 1998 until 2009, and acquired for this manuscript [11]. The supplementary data spanned the years from 1995 to 1999 and only described prescription counts at the individual drug level for all of the cephalosporins, macrolides, and quinolones, as well as prescription counts at the antimicrobial group level for the tetracyclines and broad spectrum penicillins. Both datasets are based upon the CSC dataset, which is developed by accessing all marketed outpatient drug data dispensed via prescriptions by 5900 geographically representative retail pharmacies across Canada, with provincial level coverage ranging from 51% to 88%. A patented geospatial extrapolation is used to infer use across all 8800 pharmacies (current to May 2013) in order to compensate for non-reporting pharmacies. It is based on the principle that geographically close points will have similar values. IMS Health Canada has conducted studies validating that stores in close proximity tend to have the same prescription volumes. The extrapolation stratifies by pharmacy size, type, and by province [12]. This extrapolation methodology considers the number of stores in the geographic area, distance between stores, and store size, thereby nullifying any variance in the store coverage over time and across geographic areas allowing for accurate comparisons and trending of prescriptions and unit volumes across the country. All data were reported monthly by province for all new and refilled prescriptions. The 2000 to 2010 CSC dataset similarly included individual drug level prescription count information, and also included manufacturer name, extended units prescribed (total number of tablets, capsules, millilitres, etc.), drug strength, volume of active ingredient, and cost of the prescription (total paid by the patient and/or insurer).

Data from Newfoundland and Prince Edward Island were provided as combined values for the years 1999 to 2004. In 2005 and subsequent years, data from these provinces were provided individually.

Tables containing data for prescriptions per 1000 individual-days and Defined Daily Doses per 1000 individual-days presented here can be found in the CIPARS Human Antimicrobial Use Short Report (2000–2010) and are available upon request from the corresponding author. Based on these data, readers can re-create the dataset used to carry out the analysis presented in this study.

Oral antimicrobials of the World Health Organization’s (WHO) J01 antimicrobial therapeutic classification (ATC) group (anti-infectives for systemic use) were retained for assessment [13]. Information regarding orally administered vancomycin (ATC group A07AA) was included in the analysis under class J01XA.

### Data Analysis

Four measures of consumption were used for analysis: Prescriptions per 1,000 Individual-Days (PrIDs), Defined Daily Doses per 1000 individual-days (DIDs), Defined Daily Doses per prescription (DDDs per prescription), and dollars spent per 1000 individual-days. PrIDs represent the number of prescriptions for oral antimicrobials that were dispensed through community pharmacies to a population within a defined geographic area on a daily basis. Similarly, DIDs represent an estimate of the standard doses of antimicrobials dispensed in the Canadian population by community pharmacies. Since individual level prescribing data are lacking in Canada, DDDs per prescription facilitate the estimation of changes in the dosages and/or length of prescriptions, in the absence of this specific detailed information. The dollars spent per 1000 individual-days represent the total cost of the prescriptions dispensed through the community pharmacy and includes the cost of the prescription, the pharmacy mark-up as well as the dispensing fee, regardless of whether the patient was privately or publically insured. As data regarding the extended units, drug strength, and cost were not available for data from 1995 to 1999, only the PrID measure could be calculated for this specific time frame. Population size estimates were extracted from updated and preliminary post-census data compiled by Statistics Canada [14].

To calculate the DID measure, the total Defined Daily Doses (DDDs) were first calculated for each antimicrobial for the 2000–2010 data: the volume of active ingredient was first calculated by multiplying the number of extended units dispensed by the strength of the product (measured in grams). The volume of active ingredient was then divided by the 2012 DDD value provided by the WHO [13]. In cases where a DDD value was not yet approved, the temporary DDD value posted on the WHO website was used [13]. When combination drugs were evaluated, the active ingredient volume was summed for all antimicrobial ingredients, and the volume was divided by the DDD value for the active ingredient used to calculate the DDD. Finally, the DID value was calculated by dividing the total number of DDDs for each antimicrobial (or group) by the size of the appropriate population in thousands and by the number of days in the respective year.

Similarly, the PrID and dollars spent per 1000 individual-days measures were calculated by dividing the total number of prescriptions (or dollars spent) by the population in thousands and by the number of days in the respective year. Correction factors for inflation were acquired from the Bank of Canada (http://www.bankofcanada.ca/rates/related/inflation-calculator/), and all spending data were adjusted to reflect the value of the dollar in 2010. Finally, the DDDs per prescription measures were calculated by dividing the DID metric by the PrID metric.

### Statistical Methods

Linear mixed models were built to assess differences in overall use over time for each of the four measures while accounting for repeated measures. Year and its quadratic term (where appropriate to model a curvilinear relationship) were assessed as predictors for antimicrobial use at a p value of <0.05. A linear mixed model describing the PrIDs was also built for the data from 1995 to 2010 at the antimicrobial group level. Year, antimicrobial group, a quadratic term for year, and interaction terms between group and year, and group and the quadratic term for year (where appropriate) were assessed as predictors at a p value of <0.05. Inclusion of quadratic terms allows the modelling of the curve of the data over time more accurately. Without this term, the data would show increases or decreases in a linear fashion over time, which would be misleading as graphing of the raw data showed a non-linear trend. The inclusion of interaction terms allowed the examination of other variables that might affect the trends of antimicrobial use over time. In all models, repeated measures were reconciled by assigning a covariance structure to the residuals, and the best fitting covariance structure was chosen using the most negative Akaike information criteria. Heteroskedasticity of the residuals was assessed visually, and where necessary, corrected using the natural logarithm, square root or inverse transformations of the outcome variables. Normality was assessed by consensus using a combination of normality tests at p<0.05. Outlying observations were assessed by standardized residuals. Where extreme observations were present, models were re-run without these observations to assess their impact on the model parameters. Data for these extreme observations were assessed to assure that recording errors were not present. Predictions were back-transformed in order to produce graphics.

To assess the impact of prescribing to children upon the average DDDs per prescription, linear models were built to describe the proportion of liquid antimicrobials out of all total prescriptions for the broad spectrum penicillins and cephalosporins; two classes of antimicrobials likely to be prescribed to children.

European antimicrobial use data from 2009 were acquired from ESAC-Net, and rankings performed such that the country with the lowest use was assigned a rank of 1. All calculations and analyses were performed using SAS 9.3 software for Windows © 2010 (Cary, NC, USA) and graphs produced in Microsoft Office Excel 2007 © 2010 (Redmond, WA, USA).

## Results

All antimicrobials for which data were available were classified into ten ATC codes and then sorted into eight groups: (1) broad spectrum penicillins, (2) cephalosporins, (3) sulfonamides and trimethoprim, (4) macrolides, lincosamides, and streptogramins, (5) narrow spectrum penicillins, (6) quinolones, (7) tetracyclines, and (8) “other” antimicrobials. The antimicrobials within these groupings are listed in Table 1. Not all antimicrobials were prescribed in all provinces in all years.

**Table 1:** Groupings of oral antimicrobials dispensed in Canadian outpatient pharmacies 2000–2010 based on the WHO Antimicrobial Therapeutic Classification system (12).

ATC Classification	Antimicrobial
Broad spectrum penicillins	Amoxicillin, amoxicillin with enzyme inhibitor, ampicillin carbenicillin, pivampicillin
Cephalosporins	Cefaclor, cefadroxil, cefixime, cefprozil, cefuroxime axetil, cephalexin, cephradine
Macrolides, lincosamides, streptogramins	Azithromycin, clarithromycin, clindamycin, erythromycin, lincomycin, spiramycin, telithromycin
Narrow spectrum penicillins	Bacampicillin, cloxacillin, dicloxacillin, flucloxacillin, penicillin G, penicillin V, pivmecillinam
Other antimicrobials	Chloramphenicol, erythromycin-sulfisoxazole, fosfomycin, fusidic acid, kanamycin, linezolid, methenamine, metronidazole, neomycin, nitrofurantoin, tobramycin, vancomycin
Quinolones	Ciprofloxacin, gatifloxacin, gemifloxacin, grepafloxacin, levofloxacin, moxifloxacin nalidixic acid, norfloxacin, ofloxacin, trovafloxacin
Sulfonamides and trimethoprim	Sulfadiazine, sulfadiazine and trimethoprim, Sulfamethizole, sulfamethoxazole, sulfamethoxazole and trimethoprim, sulfapyridine, sulfisoxazole, trimethoprim
Tetracyclines	Demeclocycline, doxycycline, minocycline, tetracycline

**Figure 1:**
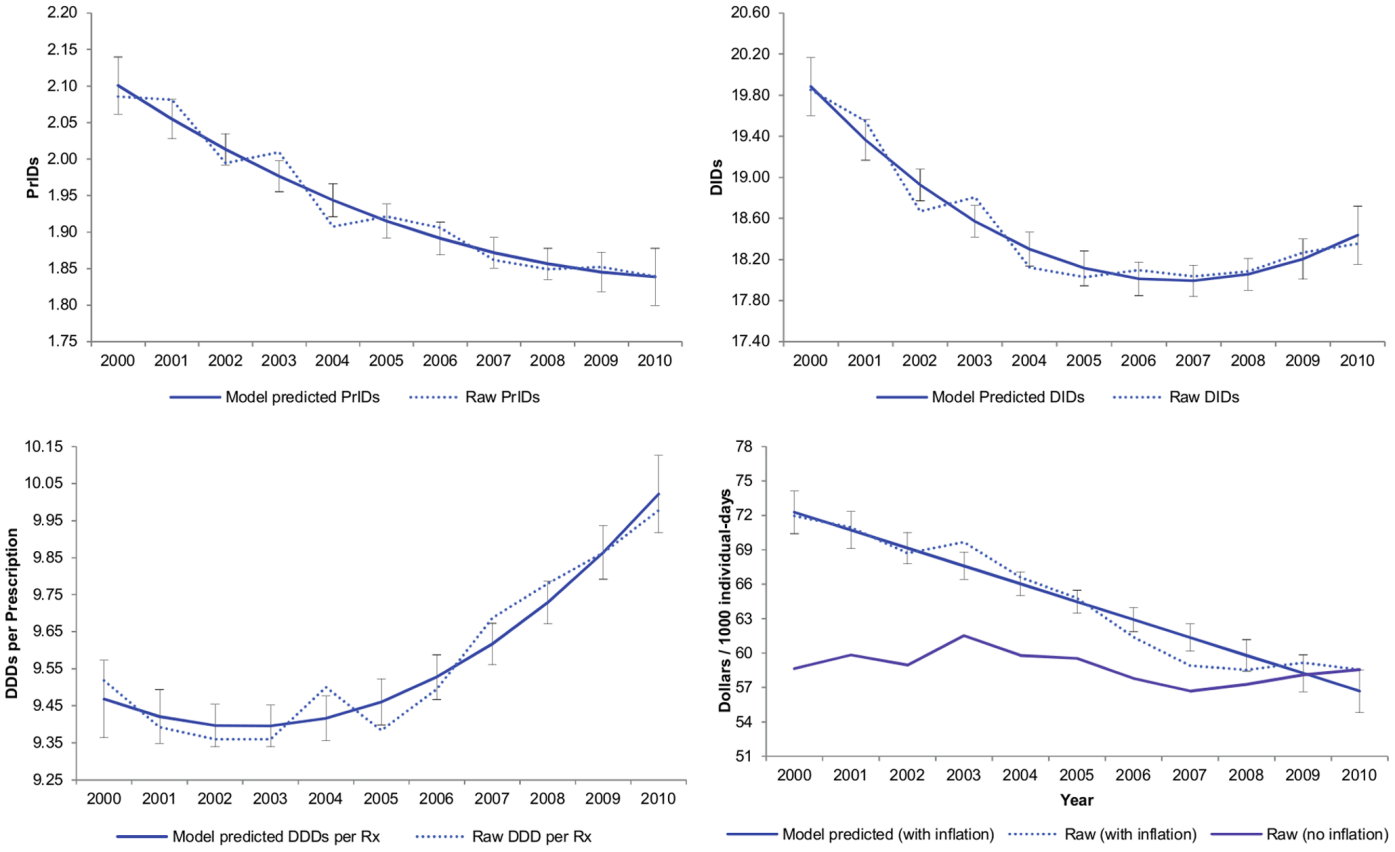
Raw and linear model PrIDs, DIDs, DDDs per prescription, and inflation-adjusted dollars spent per 1000 individual-days for antimicrobial drugs, with 95% confidence limits, dispensed by outpatient pharmacies in Canada (2000 to 2010).

Year was a significant predictor for the models built to describe overall use by the PrID, DID, DDD per prescription, and inflation-adjusted dollars per 1000 individual-days measurements. The quadratic term for year was also significant for all models with the exception of the dollars per 1000 individual-days model. A simple correlation structure was found to best fit the data in all four models. Raw data and predicted values for these models are displayed in Figure 1.

**Figure 2:**
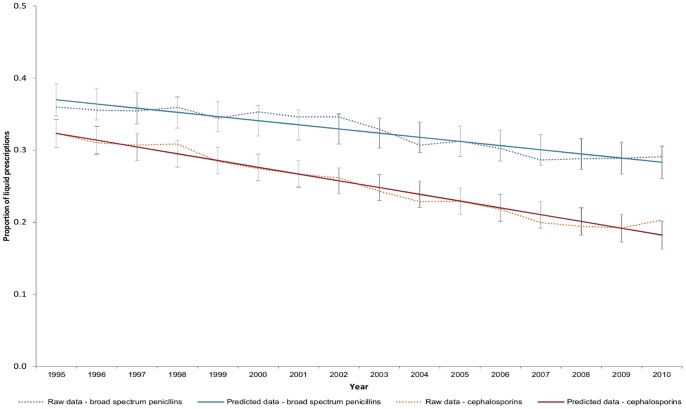
Raw data and linear mixed model predictions with 95% confidence intervals for the proportion of oral solution broad spectrum penicillin and cephalosporin prescriptions in Canada (1995 to 2010).

For the PrIDs, a year to year reduction was seen over time, with a very modest leveling off occurring during the years between 2007 and 2010. A similar decline was seen for the DIDs; however, the leveling off occurred between 2005 and 2008, and was followed by a slight increase between 2008 and 2010. The inflation-adjusted dollars spent per 1000 individual-days displayed a steady decline year to year, resulting in an overall reduction of $12 per 1000 individual-days between 2000 and 2010 (an approximate reduction of $13.1 million per year). In contrast, an increase in the average DDDs per prescription was seen from 2002 to 2010 (Figure 1).When assessing the impact of prescribing to children upon the average DDDs per prescription, a significant reduction in the proportion of liquid antimicrobial prescriptions was observed to have occurred between the years of 1995 and 2010 (Figure 2).

**Figure 3:**
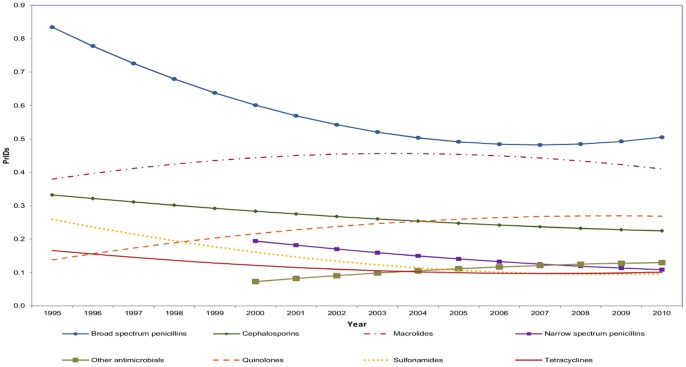
Linear model predictions for prescriptions per 1000 individual-days from 1995 to 2010 for oral antimicrobial prescriptions dispensed by outpatient pharmacies in Canada, by antimicrobial group.

Significant differences were found at the antimicrobial group level. Year and its quadratic term (year*year), antimicrobial group, interaction between the antimicrobial group and year (group*year) and interaction between antimicrobial group and the quadratic term of year (group*year*year), were found to be significant predictors for the PrIDs between 1995 and 2010, all at p<0.001. The most dramatic pattern observed was that for the broad spectrum penicillins, which declined rapidly between 1995 and 2006, followed by a plateau from 2007 to 2010 (Figure 3). Contrasts of all pairwise combinations revealed significant differences in amounts of antimicrobials dispensed among the majority of antimicrobial groups each year. Non-significant differences were found among the sulfonamides, tetracyclines, and “other” antimicrobial groupings, likely related to their low level of prescribing in comparison to the penicillins, cephalosporins, and macrolides.

Using Canadian PrID, DID, and DDD per prescription values for 2009, antimicrobial use in Canada was ranked against use reported by European countries (Table 2) participating in ESAC-Net (10). When ranked with the 32 European countries by the DID measure in 2009, Canada was 15^th^ (lowest use was ranked 1^st^, highest 33^rd^). Similarly, when ranked with the 17 countries reporting PrIDs in 2009, Canada was 5^th^ of 18. In contrast, when ranked by DDDs per prescription, Canada was ranked much higher: 16^th^ of 18^th^.

**Table 2:** Comparison of total antimicrobial use among Canada and the reporting ESAC-Net countries by DID, PrID, and DDD per prescription measures in 2009.

	DID		PrID		DDD per prescription	
Country	Value	Rank	Value	Rank	Value	Rank
Austria	15.9	10	2.0	7	8.1	11
Belgium	27.5	28	2.5	10	10.9	17
Bulgaria	18.6	17	3.3	15	5.7	3
**Canada**	18.2	15	1.8	5	9.9	16
Croatia	21.2	22	2.9	13	7.4	8
Cyprus	34.4	32	NR	NR	NR	NR
Czec Republic*	18.4	16	2.1	8	8.9	12
Denmark	16.0	11	1.7	4	9.3	14
Estonia	11.1	3	1.7	3	6.5	4
Finland	18.0	14	1.9	6	9.5	15
France	29.6	31	NR	NR	NR	NR
Germany	14.9	8	NR	NR	NR	NR
Greece	38.6	33	5.3	17	7.3	7
Hungary	16.0	12	NR	NR	NR	NR
Iceland	19.3	18	NR	NR	NR	NR
Ireland*	20.8	21	2.7	12	7.8	10
Israel	22.4	24	NR	NR	NR	NR
Italy†	28.7	30	11.0	18	2.6	1
Latvia	10.5	2	NR	NR	NR	NR
Lithuania	19.7	20	3.0	14	6.6	5
Luxembourg	28.2	29	NR	NR	NR	NR
Malta	21.6	23	NR	NR	NR	NR
Norway	15.2	9	NR	NR	NR	NR
Poland	23.6	26	NR	NR	NR	NR
Portugal	22.9	25	2.5	11	9.0	13
Romania	10.2	1	NR	NR	NR	NR
Russian Federation	12.2	5	4.1	16	3.0	2
Slovakia	23.8	27	NR	NR	NR	NR
Slovenia	14.4	7	2.1	9	6.8	6
Spain	19.7	19	NR	NR	NR	NR
Sweden	13.9	6	1.2	1	11.8	18
The Netherlands	11.4	4	1.5	2	7.4	9
United Kingdom	17.3	13	NR	NR	NR	NR

(Lowest use ranking = 1).

NR = Not reported; DDD = Defined Daily Doses; PrID = Prescriptions per 1,000 Individual-Days;

* 2008 values.

† 2007 values.

## Discussion

Our analysis of overall antimicrobial use in outpatient settings in Canada for the years 1995 to 2010 displays some modest success for stewardship stakeholders and potential areas for future research and continued improvement. The use of multiple measures allowed for a more complete picture of prescribing, use and associated costs than any single measurement alone. Significant decreases in the population-adjusted prescriptions (12.5%) and Defined Daily Doses (7.3%) from 2000–2010 suggest that Canadian prescribers have reduced the use of antimicrobial agents within the outpatient population. Spending on antimicrobials was also reduced over time, by an approximate $13.1 million per year from 2000 to 2010.

Overall, the main metric that is increasing over time is the defined daily doses per prescription, which aims to provide an estimate of changes happening in the levels of dosage or length of prescription being dispensed through community pharmacies. The increase observed in the average number of DDDs per prescription coupled with a decrease in the proportion of liquid antimicrobial prescriptions reflects the changing population in Canada. Due to lack of complete age information in the dataset, it was assumed that liquid prescription information predominantly represented use by children and tablets prescriptions predominantly represented use by adults, and used this information to determine if changes of DDDs per prescription were being observed due to changing prescription practices following new guidelines for treatment of children. The proportion of liquid broad spectrum penicillin and cephalosporin prescriptions decreased between 1995 and 2010, suggesting that prescribing for children relative to prescribing for adults decreased over the time frame examined, which would result in an increase in the average DDDs per prescription. However, what was observed when looking at the demographics of the Canadian population during the study period is that there has been a decrease in the proportion of the population consisting of children (less than 10 years of age) and an increase in the proportion of the population over the age of 65 (data not shown). This observation could offer an explanation for the increase observed among DDDs per prescription, as prescriptions for adults are expected to have a higher total number of DDDs per prescription as compared to prescriptions for children. Children’s dosing requirements are often based on weight, resulting in less active ingredient being delivered to a child than an adult over the course of an antimicrobial treatment [15]. Therefore, the number of DDDs in a child’s prescription is likely to be less than the DDDs of an adults prescription. Nonetheless, within Canada physicians are now providing less antimicrobial prescriptions overall and those that are provided may be at a higher dosage and/or for a longer period of time, which has been advocated in several guidelines. Unfortunately, data is not available to us at this time to confirm the use of higher doses or more prolonged prescribing times. In order to monitor the impact of population changes on antimicrobial use in the absence of age-specific prescribing data, we suggest that the average DDDs per prescription may be a more accurate measure, not only for assessing population changes, but also for identifying true trends in antimicrobial use when interpreted in the context of changing population dynamics.

An overall reduction in the proportion of prescriptions for broad-spectrum prescribing and smaller reductions in the prescriptions for cephalosporins, sulfonamides, and tetracyclines were highlighted in this report. As broad spectrum agents are more likely to select for pathogens with antimicrobial resistant traits, narrower spectrum agents are a favourable choice when the pathogen of interest is known [16]. However, increases in the use of the quinolone, macrolide, and “other” antimicrobial groups remain a concern for the potential for increased selection of resistant pathogens. There have been many published reports presenting evidence of the increase of quinolone resistant infections around the world, not only in organisms causing hospital-acquired infections, but also among food-borne organisms [17]–[21]. In particular, some of these reports have also demonstrated a link to prior use of this class of antimicrobials [20]–[21]. In the face of increasingly prevalent reports of quinolone resistance in a number of pathogens, our results suggest that this is an area requiring further research and additional interventions to reduce inappropriate use.

The secondary objective of this report was to determine how Canada ranks against other countries in terms of antimicrobial use. DID and PrID data suggested that use in Canada was comparable to that in the European countries with lower prescribing, while the DDD per prescription measure displayed that Canada was among the high usage countries. These values suggest that the average volume of antimicrobial agent dispensed per prescription is higher in Canada than other countries, which may reflect prescribing to adults rather than children. However we do not know how the age distribution compares between Canada and other EU countries. It is also unknown if prescribing practices are different for similar medical conditions. Measures of the proportion of prescriptions for children and age distribution comparisons may be required in order to successfully compare use in Canada to European countries for this metric.

We acknowledge the limitations of our study, which include the lack of complete data for the 1995 to 1999 time period and the potential for non-representativeness of measured pharmacies. However, despite missing an overall measure of the prescribing rates between 1995 and 2010, the prescription information for groups other than the narrow spectrum penicillin and other antimicrobials display sufficient information to describe trends in use over time. Furthermore, the large proportion of pharmacies represented in the dataset (>67% of the Canadian pharmacy universe in May 2013), and the extensive extrapolation method used by IMS Health Canada supports the contention that this data accurately reflects antimicrobial use patterns in Canada [1], [12].

Continued efforts to focus on antimicrobial use surveillance and stewardship are strongly advised, including improving compliance with the continued use of published prescribing guidelines regarding first and second line choices, with appropriate dosing and duration. Furthermore, these guidelines should emphasize discontinuing therapy once the recommended treatment duration is complete. Public education surrounding the effective and appropriate use of antimicrobials should be improved, describing the role that inappropriate use has on the development of antimicrobial resistance, potential treatment failures and collateral damage to the normal flora. A multifaceted approach is required in order to change the social environment surrounding antimicrobial use in Canada.
